# Chemical composition, physical properties, and immunomodulating study of mare's milk of the Adaev horse breed from Kazakhstan

**DOI:** 10.3389/fnut.2025.1443031

**Published:** 2025-04-28

**Authors:** Gaukhar Kossaliyeva, Kaster Rysbekuly, Karlygash Zhaparkulova, Sabira Kozykan, Juxiu Li, Assiya Serikbayeva, Zhanserik Shynykul, Maira Zhaparkulova, Zura Yessimsiitova

**Affiliations:** ^1^College of Food Science and Engineering, Northwest A & F University, Xianyang, China; ^2^Department of Biotechnology, Faculty of Biology and Biotechnology, Al-Farabi Kazakh National University, Almaty, Kazakhstan; ^3^School of Pharmacy, S.D. Asfendiyarov Kazakh National Medical University, Almaty, Kazakhstan; ^4^Department of the Technology of Food Products and Food Safety, Faculty of the Zooengineering and Food Production Technology, Kazakh National Agrarian Research University, Almaty, Kazakhstan; ^5^Higher School of Medicine, Al-Farabi Kazakh National University, Almaty, Kazakhstan; ^6^Pulmonary Therapy Department, National Scientific Center of Phthisiopulmonology, Almaty, Kazakhstan; ^7^Department Biodiversity and Bioresources, al-Farabi Kazakh National University, Almaty, Kazakhstan

**Keywords:** mare's milk, chemical composition, physical properties, immunomodulating properties, histology

## Abstract

Mare's milk is recognized for its nutritional and immunomodulatory properties, making it a promising functional food. Furthermore, mare's milk is characterized by anti-carcinogenic and antiviral attributes, which have incited considerable scientific inquiry. This study investigates the chemical composition, immune-modulating effects, and physiological impact of Adaev horse milk supplementation in a *Streptococcus pneumoniae*-infected Wistar rat model. Eighteen male rats were divided into three groups: a control group (GC-1) receiving standard chow, a low-dose supplementation group (LDM-2) receiving 0.1 g of lyophilized mare's milk, and a high-dose group (HDM-3) receiving 1.5 g of lyophilized mare's milk. SDS-PAGE analysis revealed that Adaev horse milk is rich in whey proteins and has lower casein content, enhancing protein digestibility and bioavailability. HILIC-MS identified key sialylated oligosaccharides [lactose, 3′-sialyllactose (3′SL), 6′-sialyllactose (6′SL), 3′-α-sialyl-N-acetyllactosamine (3′SLN), sialyllacto-N-tetraose a (LSTa), sialyllacto-N-tetraose b (LSTb), and sialyllacto-N-tetraose c (LSTc)], suggesting potential prebiotic and immunomodulatory effects. Blood serum analysis demonstrated increased total protein levels in supplemented groups, with significant alterations in albumin/globulin ratios, creatinine, and enzyme activity. Histological examination of lung tissues indicated that high-dose supplementation reduced inflammatory damage, improved tissue integrity, and enhanced immune recovery. These findings suggest that Adaev horse milk supplementation modulates immune responses, improves metabolic and hematological parameters, and mitigates pneumonia-induced tissue damage, highlighting its potential as a functional dietary supplement with immunotherapeutic benefits.

## 1 Introduction

The relationship between diet and health underscores the importance of functional food research. Kazakhstan has significant potential in this area, particularly with dairy products like mare's milk (1). Dairy items are nutritionally valuable (2), versatile in cooking (3), and beneficial for digestive health due to probiotics in products such as yogurt and kefir (4, 5). Among dairy options, mare's milk stands out for its unique nutritional benefits, despite being less commonly consumed than cow's or goat's milk (6).

Mare's milk is a nutritionally rich dairy product containing essential biological elements, including amino acids, whey proteins, lipids, and enzymes such as lysozyme and amylase, along with vital microelements like calcium, potassium, phosphorus, magnesium, and iron (6–9). It provides significant amounts of vitamins A, C, B-complex, E, H, and folic acid in well-balanced proportions, contributing to its high nutritional value (7). Chemically, mare's milk consists of ~89.7% water, 2.3% protein, and notable concentrations of ascorbic acid, phosphorus, potassium, magnesium, and calcium, often surpassing levels found in other livestock milk (6, 10). Despite its relatively lower protein content compared to cow's or goat's milk, it contains more protein than human milk, making it a suitable alternative in neonatal nutrition (6, 7). The primary whey proteins in mare's milk include β-lactoglobulin, α-lactalbumin, immunoglobulins, lactoferrin, and lysozyme, with a protein profile characterized by lower β-lactoglobulin and higher α-lactalbumin and immunoglobulin levels compared to cow's milk, providing enhanced thermal stability (11–14). Casein fractions, including αs1–, αs2–, β-, and κ-casein, exist in micellar structures that store essential amino acids and minerals, playing a crucial role in foal nourishment (15). Additionally, mare's milk has higher ascorbic acid levels than cow's or goat's milk but lower concentrations of phosphorus, potassium, magnesium, and calcium, though its calcium-to-phosphorus ratio is significantly higher than that of cow's or goat's milk but lower than that of human milk (16). A study of 29 lactating mares revealed that colostrum contained 16.41% total protein, 13.46% whey protein, and 2.95% casein, with non-protein nitrogen levels increasing post-birth, highlighting its evolving nutritional composition during lactation (17–19). While its amino acid profile resembles that of ruminant milk, it is particularly rich in cysteine, glycine, serine, and glutamic acid but has lower methionine levels, contributing to its distinctive nutritional properties (19–25). The physicochemical characteristics of mare's milk vary with diet, health status, and lactation stage, with its pH gradually increasing from 6.6 at 4 days post-birth to 6.9 at 20 days and reaching 7.1 by day 180, primarily influenced by protein and salt concentrations (26, 27). Furthermore, its freezing point (−0.554°C) is lower than that of cow's milk due to its higher lactose content, with a typical pH range of 7.1 – 7.3 and an average freezing point of −0.525°C (28). Hygienic standards for dairy products emphasize total bacterial and somatic cell counts as key indicators of milk quality and safety, ensuring compliance with national and international regulations to safeguard human health (29, 30).

Bacterial infections are caused by pathogenic bacteria that can enter the body and multiply, leading to various health issues. Some common bacterial infections in humans include streptococcal infections, staphylococcal infections, *E. coli* infections, and many others. Among them, *Streptococcus pneumoniae* is a type of bacterial infection that can affect humans (31). The symptoms and severity of bacterial infections can vary depending on the type of bacteria involved and the site of infection. Horse milk, also known as mare's milk, has been consumed by some cultures for centuries and is believed to have certain health benefits for a wide range of infections, including pneumonia, sinusitis, ear infections, and invasive diseases like bacteremia and meningitis. However, horse milk consumption during infection, like any dietary choice, should be approached with caution and under the guidance of a healthcare professional. While horse milk is consumed in some cultures and is believed to have certain health benefits, there is limited scientific evidence to support its specific role in treating or preventing infections.

The Adaev horse is an indigenous and distinct horse breed that has its roots in the Kazakh Adayev tribe, which historically lived in the lowlands around the Aral-Caspian Sea region. This particular breed of horse plays an irreplaceable role in the challenging and rugged environments of the Ustyurt plateau and the Mangistau Peninsula, where its unique characteristics make it well-suited to thrive (32). Throughout millennia, people in Central Asia and neighboring regions have incorporated Adaev horse milk into their diets. This milk serves as a fundamental ingredient in the production of a traditional beverage called qymyz, which is crafted through a fermentation process that involves lactic acid bacteria and yeasts. Also, its derivatives such as dry powder and others serve as functional foods and food supplements. Qymyz holds immense cultural significance, playing a central role in the traditional horse husbandry practices and the cultural identity of the Central Asian population. It symbolizes good health, and besides its cultural importance, mare's milk, including qymyz, has been reported to offer various therapeutic benefits. In this particular study, the focus was on the physico-chemical properties of horse milk, with a specific emphasis on milking and sample collection from the Adaev horse breed. The primary objective of this research is to investigate the immunomodulating properties of Adaev horse milk. This research has the potential to contribute to our understanding of the immunomodulating properties of mare's milk and its potential applications in combating multidrug-resistant bacterial strains, such as *S. pneumonia*.

## 2 Materials and methods

### 2.1 Chemicals, reagents and strains

One of the testing bacterial strains of the American-type collection center (ATCC) culture was used as a marker strain in the current study, which is *S. pneumoniae* 79. 90% alcohol and xylene were purchased from Sigma Aldrich.

### 2.2 Animals and experimental design

Animals: Ethical approval was obtained from the Committee of Animal Care and Use, Al-Farabi Kazakh National University, Kazakhstan. Eighteen male adult Wistar rats (5 weeks old) were housed in the animal facility of the Biology and Biological Technologies Faculty at Al-Farabi Kazakh National University. The environmental conditions were strictly controlled, maintaining a 12 h light/dark cycle and an ambient temperature of 23°C. Rats had unrestricted access to food and water, and their cages were cleaned daily and disinfected weekly with 70% alcohol. They underwent a seven-day acclimatization period with free access to water and rat chow (HFK Bioscience Co., LTD, Beijing, China).

**Figure 1:**
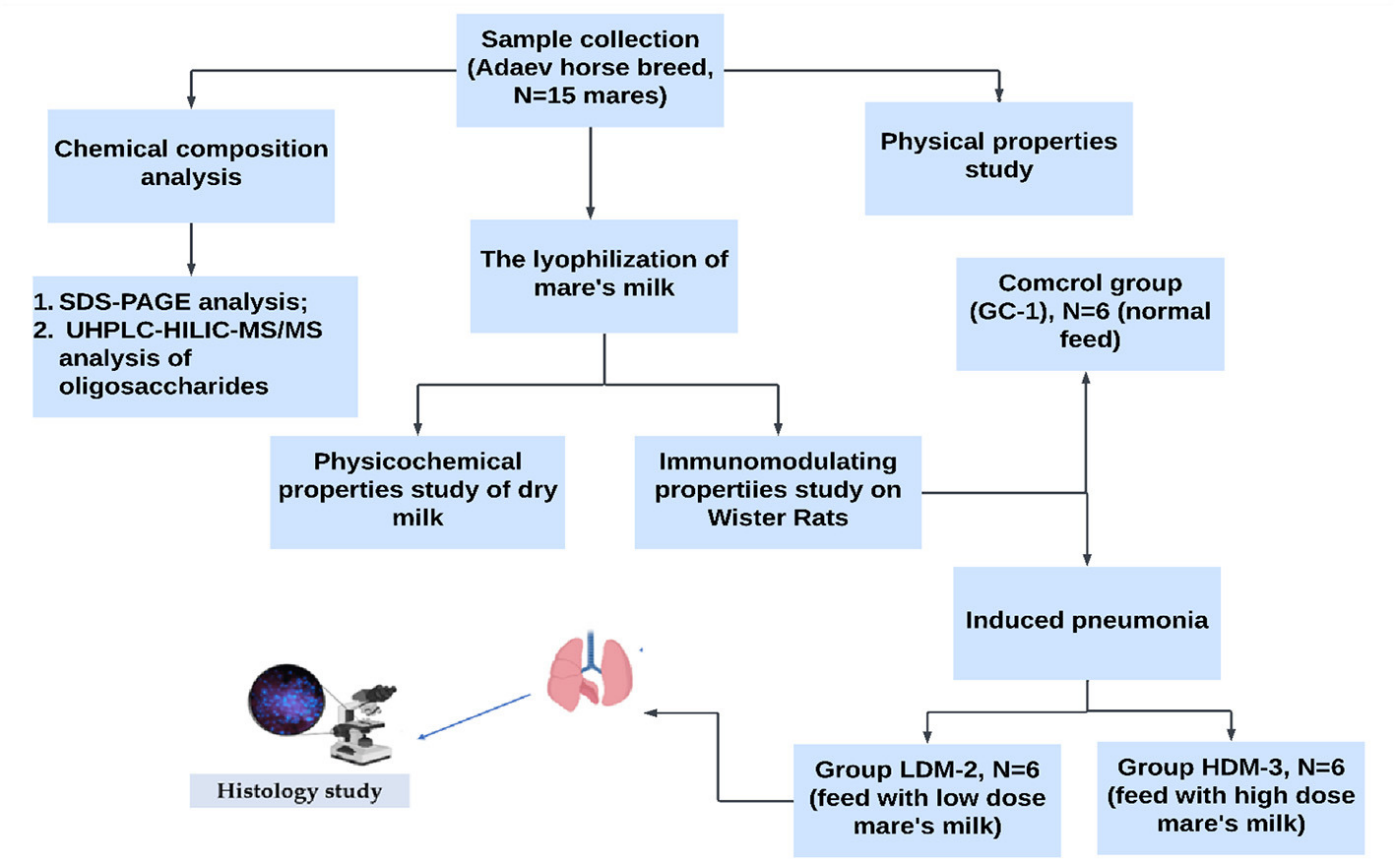
Experimental design for investigating the immunomodulating properties of mare's milk.

Experimental Design: The study followed a structured experimental design, including sample collection, physicochemical analysis, lyophilization, assessment of the immunomodulatory properties of mare's milk, blood and blood serum analysis, and histological examination. The experimental design is illustrated in Figure 1.

Eighteen male Wistar rats (200 ± 1.2 g) were randomly divided into three groups (*n* = 6 per group). The control group (GC-1) received only rat chow (4.5% fat, 0.02% cholesterol) and water. The low-dose mare's milk group (LDM-2) received rat chow supplemented with 0.1 g of lyophilized mare's milk powder, while the high-dose mare's milk group (HDM-3) received rat chow supplemented with 1.5 g of lyophilized mare's milk powder. After acclimatization, LDM-2 rats were injected with 0.3 mL (1,000 CFU) of a mixed strain of *S. pneumoniae* 79, while HDM-3 rats received 1.5 mL (1,000 CFU) of the same strain.

To account for individual differences in initial body weight, baseline weight was recorded and used as a covariate in the statistical analysis to adjust for potential variations among the groups.

### 2.3 Mare's milk sampling

During the research period, three samplings of Adaev mare's milk were carried out in Almaty, Kazakhstan. Samples of mare's milk were taken during March and August 2023 in various stages—from the first to the sixth month of lactation. The rats were housed individually, both indoors and outdoors, and received a daily diet consisting of wheat bran, grass, and hay. Access to water was provided continuously through troughs. Mares were between 5 and 9 years of age, with live weights between 565 and 790 kg. The minimum quantity of milk samples from each mare was about 200 mL. The collected number of milk samples was comparatively small as it is very difficult to take milk samples from mares if foals are not in their immediate vicinity (but without physical contact and the possibility to suck milk). Since foals suck quite often, mostly every 15 min, it is very difficult to gather a higher milk quantity by one milking, which would be sufficient for a great number of analyses.

### 2.4 Chemical composition and physical properties analysis

Samples of mare's milk for the analysis of chemical composition and physical properties were collected from a total of 15 mares. The analyses of milk chemical composition included determining the content of total solids, milk fat, proteins, caseins, and lactose. All mentioned analyses were determined by reference methods in order to calibrate the instrument MilkoScan FT 2 (Foss Electric, Denmark) (33). The content of whey protein and non-protein nitrogen in milk was determined by calculating the difference in the total protein and casein content. Among physical properties, titratable and ionometric acidity, as well as the freezing point of mare's milk were determined. The titratable acidity of milk was carried out according to the Soxhlet-Henkel method and the pH value of milk was by ionometric method. Determination of the physicochemical mass of the mare's milk was done before and after the lyophilization step.

### 2.5 Lyophilization

The freeze-drying process was carried out on a semi-technical scale using the CHWC-20A freeze-dryer (Lyo-Tech Sp.z o.o., Miedzyrzecz, Poland) to simulate an effective industrial process. Freeze-drying occurred in a single layer of frozen milk discs. Throughout the process, the vacuum was kept approximately constant in the range of 60–70 Pa, and the temperature of the heating plates was varied, starting at 90°C and ending at 40°C. The complete freeze-drying process was conducted for 16 h. The powders were stored in foil laminate sealed pouches at 3 ± 0.5°C for further analyses, but not for longer than 2 weeks. Freeze-dried mare's milk samples were stored and protected from light (34).

Dry matter (DM) was determined according to the AOAC standard procedure (AOAC, 1990). Contents of dry matter were calculated according to the following equation DM (%) = m_2_ × 100/m_1_, where DM (%) is the percentage of a total dry matter, m_1_ is sample weight before DM determination and m_2_ is sample weight after drying. Overall, the obtained powder mass was 2 kg from 45 L of mare's milk.

### 2.6 Body weight of animals

The body weight of each rat was recorded at baseline (before treatment) and measured weekly until the end of the study. A large plastic beaker was placed on an electronic weighing scale, tared to zero, and then removed. Each rat was placed inside the beaker, which was repositioned on the scale, and the most frequently observed weight was recorded (33). To ensure an accurate assessment of treatment effects, initial body weight was incorporated as a covariate in the statistical analysis. By the end of the 5-week experiment, all rats were fasted overnight before proceeding to the next phase of the study.

### 2.7 Blood serum analysis

The blood of each mouse was collected from the inner canthus and the animals were sacrificed by neck dislocation. In blood serum of rats by using of automatic biochemical analyzer Rendom Access A-25 (Spain) were determined the following parameters: creatinine, urea, alanine aminotransferase (ALT), aspartate aminotransferase (AST), alkaline phosphatase (ALP), gamma-glutamyltransferase (GGT), total protein, albumin, albumin-globulin, and glucose. All parameters were determined using Bio Systems kits (Spain).

### 2.8 Hematological analysis

Histological processing of the material was carried out using the traditional method of microscopic technique for preparing thin slices. In the hematological analysis, profiles of both the test and control animals were examined using blood samples collected in EDTA-containing tubes. An automated hematology analyzer (OX-360, Balio Diagnostics, France) was employed for this analysis. These included an assessment of white blood cell (WBC) counts and leukocyte formula, expressed as a percentage of total WBCs. This category encompassed various measures of red blood cells (RBCs), including RBC count, hemoglobin (Hb) concentration, hematocrit (HCT) level, mean corpuscular volume (MCV), mean corpuscular hemoglobin (MCH), mean cell hemoglobin concentration (MCHC), and red blood cell distribution width (RDW). Platelet count (PLT) and mean platelet volume (MPV) were also examined as part of the hematological analysis. These hematological parameters provide valuable insights into the cellular composition and health of the blood, aiding in the assessment of the animals' overall wellbeing and the potential impact of experimental or environmental factors on their hematological status.

### 2.9 Histology analysis

Histological processing of the material was carried out using the traditional method of microscopic technique for preparing thin slices. For morphological analysis, animals were decapitated at a strictly defined fixed time between 9 and 10 a.m. The object of the study was the main population of rat lung cells. Material from the organs of control and experimental animals was taken and fixed, followed by processing for comparative histological analysis. Material from the organs of control and experimental animals was taken and fixed, followed by processing for comparative histological analysis. Morphological changes were studied in lung tissue, from which tissue fragments measuring 1 × 1 × 1 cm were cut out and intended for histological examination, followed by fixation in a 10% solution of neutral formalin. The minimum fixation period lasted for 10 days, with the fixing fluid being replaced twice during this time. Subsequently, the fixative was thoroughly rinsed in running tap water for 24 h. The tissue underwent a dehydration process using a series of alcohol solutions with increasing concentrations (ranging from 50% to absolute alcohol), followed by clearing in xylene. Subsequently, it was immersed in a solution of paraffin in xylene saturated at +37°C and then transferred to paraffin at +56°C. Finally, the tissue was embedded in a mixture of paraffin and beeswax to create paraffin blocks, from which serial sections with a thickness of 5–7 microns were cut. The sections were stained with hematoxylin-eosin. Viewing and photography of the resulting histological preparations were performed using a Leica DMLS light microscope equipped with a Leica DFS 280 digital camera. The resulting images were processed on a Pentium 4 computer (35).

### 2.10 Extraction and purification

For the extraction and purification of mare's milk oligosaccharides (MMOs), freshly milked mare's milk (2 mL) was centrifuged at 14,000 × g at 4°C for 30 min to remove fat and debris. The supernatant was carefully collected, mixed with four volumes of cold 2:1 v/v chloroform–methanol, and centrifuged again under the same conditions to precipitate proteins. The upper aqueous phase containing oligosaccharides was transferred and subjected to ethanol precipitation by adding two volumes of cold 100% ethanol, followed by overnight incubation at 4°C and a final centrifugation step (14,000 × g at 4°C for 30 min) to pellet residual proteins. The clear supernatant was evaporated to dryness using a vacuum concentrator. The dried extract was reconstituted in Milli-Q water and loaded onto a C18 solid-phase extraction (SPE) column to remove residual lipids, and the flow-through was further purified using an aminopropyl (NH2) SPE column to eliminate salts and small molecules. The final eluted fraction, enriched in oligosaccharides, was evaporated to dryness and stored at −20°C until HILIC-ESI-MS analysis. Fresh milk was preferred for the extraction process to maintain the integrity of the oligosaccharide profile, though lyophilized samples could be used with reconstitution in water before processing.

### 2.11 SDS-PAGE analysis

Proteins isolated from mare's milk were analyzed using sodium dodecyl sulfate-polyacrylamide gel electrophoresis (SDS-PAGE) to determine their molecular weight distribution. Raw mare's milk was first centrifuged at 14,000 g for 30 min at 4°C to separate fat and cellular debris. The supernatant was collected, and proteins were precipitated using two volumes of cold ethanol, incubated overnight at 4°C, and centrifuged under the same conditions. The resulting protein pellet was resuspended in phosphate-buffered saline (PBS) and denatured in Laemmli buffer containing β-mercaptoethanol before electrophoresis.

A 10% tris–glycine acrylamide gel was used for separation, and a pre-stained protein ladder with molecular weight markers (170, 130, 100, 70, 55, 40, 25, 15, 10, and 5 kDa) was loaded in one lane as a reference. The proteins were resolved under a constant voltage until optimal separation was achieved.

After electrophoresis, protein bands were fixed using a 40% ethanol and 10% acetic acid aqueous solution (v/v) and stained overnight with SYPRO Ruby protein gel stain for enhanced visualization. De-staining was performed using a 10% ethanol and 7% acetic acid solution (v/v) to remove background staining while maintaining clear protein bands. The gel was imaged using a Gel Documentation System, ensuring high-resolution protein visualization.

### 2.12 UHPLC-HILIC-MS/MS analysis of oligosaccharides

For the identification of oligosaccharides, an Ultimate 3,000 UHPLC system (Thermo Scientific) was coupled to an Orbitrap Fusion Lumos mass spectrometer (Thermo Scientific). Chromatographic separation was achieved using an ACQUITY Glycoprotein BEH Amide column (300 Å, 1.7 μm, 2.1 mm × 150 mm, Waters Corporation, Milford, MA, U.S.A.). The total run time was 65 min, with a mobile phase flow rate of 400 μL/min, and the column oven temperature was maintained at 35°C. The injection volume for each sample was 10 μL.

The mobile phase consisted of 10 mmol/L ammonium formate with 0.1% (v/v) formic acid (A) and 99.9% (v/v) acetonitrile with 0.1% (v/v) formic acid (B). The gradient elution program was as follows: 1.5 min of isocratic hold at 95% B, followed by a linear gradient reduction from 95% to 80% B over 8.5 min. The next 50 min saw a gradual transition from 80 to 50% B, followed by a 5 min wash phase, reducing B to 2%, and finally, re-equilibration at 95% B. During the column washing stage, the flow rate was adjusted to 250 μL/min.

Electrospray ionization (ESI) mass spectrometry detection was performed in both positive and negative ionization modes to analyze neutral and acidic oligosaccharides. The ion source voltage was set to ± 3.5 kV, with the capillary temperature maintained at 250°C. Sheath gas and auxiliary gas were supplied at 15 and 10 arbitrary units (a.u.), respectively.

The MS spectra were acquired using Fourier transform ion trap (FT-IT) and higher-energy collisional dissociation (HCD) fragmentation techniques. FT-IT spectra were recorded at a Normalized Collision Energy (NCE) of 35% with a Q value of 0.25, while HCD fragmentation spectra were obtained at NCE values ranging from 10 to 50% (10, 15, 20, 25, 30, 40, and 50). Each sample was analyzed in triplicate to ensure reproducibility.

Only identified oligosaccharides were reported in the results. The identification process was based on mass accuracy, fragmentation patterns, and retention times obtained from HILIC-MS/MS analysis. The detected oligosaccharides were confirmed using reference standards, literature data, and spectral matching against the NIST Tandem Spectral Library, ensuring the reliability of the identifications. The MS spectra were analyzed for characteristic fragment ions and structural features specific to neutral and acidic oligosaccharides present in the sample.

### 2.13 Statistical data processing

All results are presented as means ± SD (standard deviation). The significance of the collected data was evaluated using the General Linear Model (GLM) procedure for analysis of variance (ANOVA) to test the null hypothesis (H0). When H0 was rejected, Tukey's *post hoc* test was used for multiple comparisons among treatment groups. A *p-value* of ≤ 0.05 was considered statistically significant.

## 3 Results

### 3.1 Chemical composition and physical properties of Adaev horse breed mare's milk

Table 1 contains results considering the chemical composition, more precisely the content of total solids, milk fat, proteins, casein, lactose, and total solids non-fat. The chemical composition of mare's milk varied across the lactation months, with TS, F, P, and C levels showing fluctuations. Notably, fat and casein proportions exhibited significant differences (*p* ≤ 0.05) between months, indicating compositional changes over time. Protein levels ranged from 1.67 to 2.35%, while fat content varied from 1.25 to 2.97 g/100 g, with both parameters displaying statistical significance. L content remained relatively stable, with minor variations, while the proportion of casein in total protein (C') demonstrated substantial changes (*p* ≤ 0.05), reflecting dynamic protein composition shifts.

**Table 1:** Chemical composition of mare's milk.

**Month**	**TS, g/100 g**	**F, g/100 g**	**P, %**	**C, %**	**C', %**	**CPNPN, %**	**L, g/100 g**	**TSNF, g/100 g**
1st	10.07 ± 0.75	2.38 ± 0.79^*^	1.67 ± 0.29^*^	1.08 ± 0.43	55.17 ± 22.76^*^	0.52 ± 0.51	6.07 ± 0.5^*^	8.97 ± 0.23
2nd	10.92 ± 0.75	2.97 ± 0.79^*^	1.88 ± 0.29^*^	0.49 ± 0.43	23.52 ± 22.76^*^	1.47 ± 0.51	6.09 ± 0.5^*^	8.78 ± 0.23
3rd	10.93 ± 0.75	1.47 ± 0.79	1.99 ± 0.29^*^	0.97 ± 0.43	53.63 ± 22.76^*^	1.03 ± 0.51	6.15 ± 0.5^*^	8.79 ± 0.23
4th	11.03 ± 0.75	1.25 ± 0.79	2.35 ± 0.29^*^	0.97 ± 0.43	7.97 ± 22.76	1.65 ± 0.51	6.16 ± 0.5^*^	8.79 ± 0.23
5th	12.15 ± 0.75^*^	1.37 ± 0.79	1.67 ± 0.29	0.18 ± 0.43	8.15 ± 22.76	0.47 ± 0.51	6.25 ± 0.5^*^	8.88 ± 0.23
Mean ± SD	11.02 ± 0.75	1.71 ± 0.79	1.76 ± 0.29	0.49 ± 0.43	26.06 ± 22.76	0.96 ± 0.51	6.19 ± 0.5	8.77 ± 0.23
p-value	≤0.05	≤0.05	0.03	0.07	≤0.05	0.09	≤0.05	0.08

“^*^” significant values, “^,^” share of casein in relation to the total protein content in mare's milk; TS, total solids; F, milk fat; P, proteins; C, casein; CP-NPN, crude protein - non-protein nitrogen; L, lactose; TSNF, total solids non-fat.

The physical properties of mare's milk showed significant variations in titratable acidity (°SH) and freezing point (*p* ≤ 0.05), while pH remained relatively stable throughout lactation. Titratable acidity decreased progressively from 3.49°SH in the first month to 1.25°SH in the sixth month, indicating a reduction in acid content over time. The pH values ranged from 6.87 to 7.07, with the highest pH observed in the fourth month (*p* = 0.03), reflecting slight alkalization. The freezing point fluctuated slightly, with the most significant change in the sixth month (−0.5356°C, *p* ≤ 0.05), suggesting potential shifts in milk composition, such as lactose and mineral content. These results indicate that as lactation progresses, mare's milk undergoes a gradual decrease in acidity, a slight pH increase, and a shift in freezing point, which may influence its stability and processing properties.

### 3.2 Lyophilization results

The freeze-drying process did not alter the proportion of the milk components in the dry matter. The moisture content of the freeze-dried mare's milk was 10.3 g/kg, and its bulk density was below 0.1 g/mL. Overall, 2 kg of powder was obtained from 45 L of mare's milk.

### 3.3 Body weight of animals

**Figure 2:**
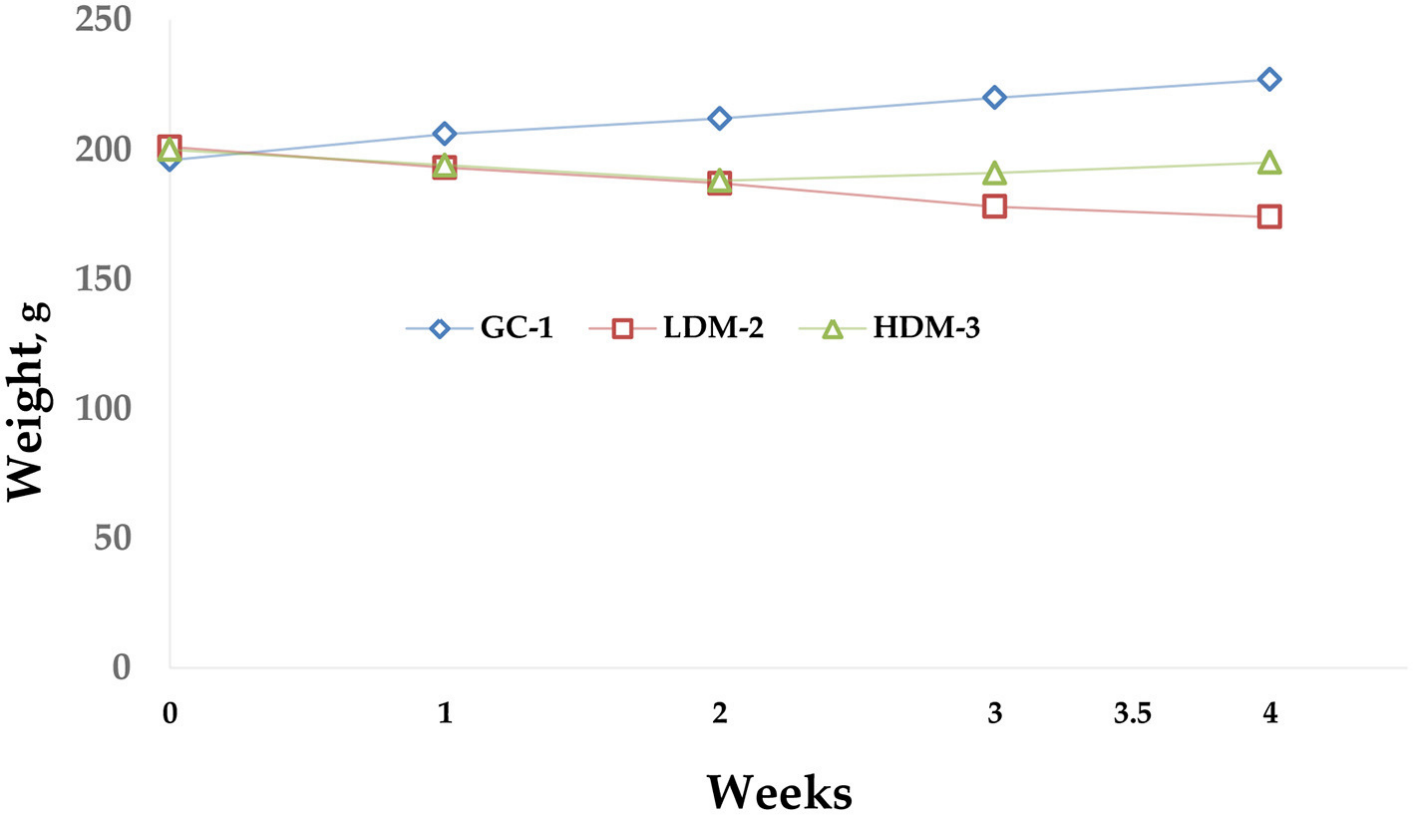
Body weight diagram. The graph illustrates the body weight dynamics of three groups of Wistar rats (GC-1, LDM-2, and HDM-3) during the experiment. The GC-1 group showed a steady increase in body weight over time. The LDM-2 group exhibited a gradual decline in weight, while the HDM-3 group demonstrated slight weight gain. Data points represent the mean weight (g) per group per week.

The diagram below (Figure 2) illustrates body weight changes over a 5-week experimental period. Overall, the HDM-3 group, which consumed 1.5 g of mare's milk, showed weight gain.

### 3.4 Physicochemical parameters after lyophilization

Freeze-drying is a known optimal drying method, especially when considering the preservation of heat-sensitive biological materials. This approach allows for the microbiological stability and long shelf life of products due to low moisture content (<2%), short reconstitution (rehydration) time due to the retention of the material's structure (low drying shrinkage), low weight, high recovery of volatiles and nutrients, and high viability and bioactivity level. According to the Table 2 results, physicochemical parameters have been slightly changed after lyophilization.

**Table 2:** Physicochemical properties of mare's milk after lyophilization.

**Item**	**SD**	**Min**.	**Max**.	***p*-*value***
Fat, %	1.92 ± 0.41^*^	0.86	2.97	≤0.05
Protein, %	1.95 ± 0.95	1.55	2.34	0.07
Solids-non-fat SNF, %	12.48 ± 4.94	10.35	14.61	0.08
Lactose, %	8.62 ± 3.78	7.26	9.97	0.09
Total solids, %	8.77 ± 5.31	8.30	9.24	0.06
Freezing point depression, m^0^C	−424.63 ± 384.61	−784.81	−1101.41	0.04^*^
Absolute freezing point, °C	−0.59 ± 0.38	−0.78	−1.10	0.05^*^
Acidity (^0^SH)	3.12 ± 2.52^*^	3.25	3.73	≤0.05
Acidity (°TH)	8.47 ± 7.12	9.53	8.26	0.07
Lactic acid, %	0.67 ± 0.06	0.61	0.73	0.08
Citric acid, %	0.18 ± 0.07	0.14	0.21	0.09
Density, g/L	1054.72 ± 20.61^*^	1043.11	1069.11	≤0.05
Urea, mg/dL	50.74 ± 21.61	44.88	56.66	0.06
Casein, %	0.61 ± 0.91	0.13	1.08	0.07

* indicates statistically significant differences (p ≤ 0.05).

### 3.5 SDS-PAGE analysis

**Figure 3:**
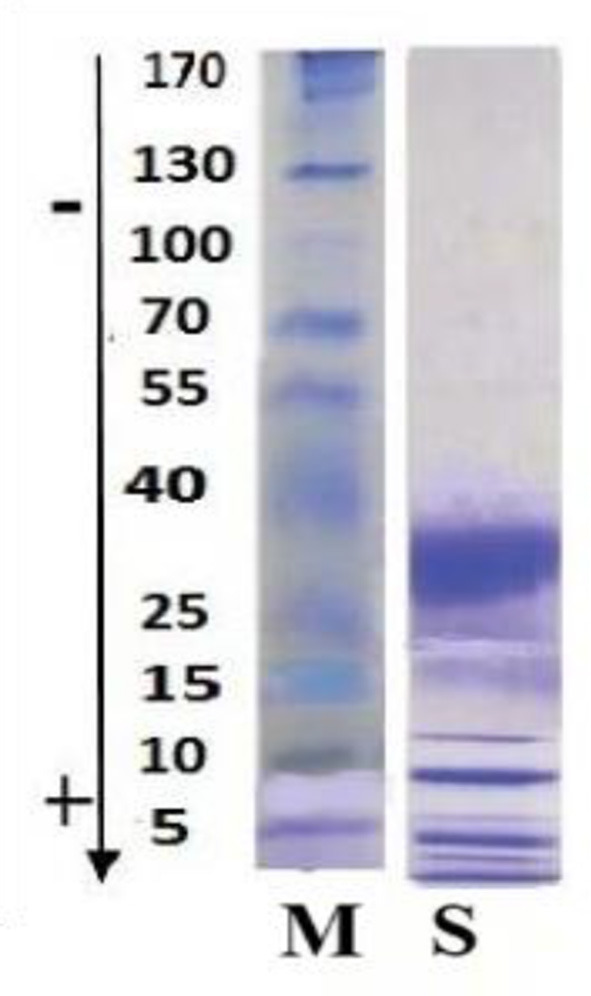
SDS-PAGE analysis of Adaev horse milk. Lane M: Molecular weight marker with protein sizes ranging from 170 kDa to 5 kDa; Lane S: Adaev horse milk protein profile, showing prominent bands around 40, 25, and 15 kDa, corresponding to β-lactoglobulin, α-lactalbumin, and serum albumin, respectively.

The SDS-PAGE analysis of Adaev horse milk (lane S) reveals a distinct protein profile with prominent bands around 40, 25, and 15 kDa, indicating the presence of key whey proteins such as β-lactoglobulin, α-lactalbumin, and serum albumin (Figure 3). The absence of high molecular weight bands (>100 kDa) suggests a lower casein content than cow's milk, aligning with the known composition of horse milk, which is rich in whey proteins and has a protein profile more similar to human milk. The presence of low molecular weight bands (~5–15 kDa) may indicate smaller peptides or bioactive proteins. Overall, the Adaev horse milk protein composition highlights its nutritional and functional potential, particularly for its high whey protein content.

### 3.6 UHPLC-HILIC-MS/MS analysis

The HILIC-MS elution profile identified lactose, 3′-sialyllactose (3′SL), 6′-sialyllactose (6′SL), 3′-α-sialyl-N-acetyllactosamine (3′SLN), sialyllacto-N-tetraose a (LSTa), sialyllacto-N-tetraose b (LSTb), and sialyllacto-N-tetraose c (LSTc) as the predominant oligosaccharides (Table 3). The separation efficiency on the HILIC column was primarily influenced by the size and polarity of the molecules. The early elution of lactose relative to sialylated oligosaccharides reflects the selectivity of HILIC, where increased polarity leads to longer retention times. The sialylated oligosaccharides exhibited higher polarity due to the presence of carboxylate ions (COO–), resulting in distinct separation patterns. The clear distinction between neutral (lactose) and sialylated (3′SL, 6′SL, 3′SLN, LSTa, LSTb, LSTc) oligosaccharides confirms the efficacy of HILIC-MS in analyzing the structural diversity of oligosaccharides in Adaev horse milk.

**Table 3:** HILIC-MS analysis of free oligosaccharides in Adaev horse milk.

**Name**	**RT**	**Theoretical m/z**	**Experimental m/z**	**Precursor type**	**Collision energy (NCE)**	**MF score**	**RMF score**
3′SL	16.0	656.2039	656.2033	[M+Na]^+^	20	899	994
6′SL	18.20	656.1980	656.1980	[M+Na]^+^	20	900	919
3′SLN	20.01	657.2340	657.2340	[M+H–H_2_O]^+^	15	992	995
LSTa	24.51	657.2354	657.2354	[M+Na]^+^	15	992	995
LSTb	25.80	1021.3330	1021.3330	[M+Na]^+^	40	857	870
LSTc	26.45	1035.4123	1035.4123	[M+Na]^+^	40	870	885

Table 3 presents the retention time (RT), theoretical and experimental m/z values, precursor ion types, collision energy (NCE), match factor (MF), and reverse match factor (RMF) scores of the free oligosaccharides detected in Adaev horse milk using HILIC-MS in positive ion mode. The identified compounds include 3′SL, 3′SLN, LSTa, LSTb, and LSTc. The precursor ions were predominantly detected as sodium adducts ([M + Na]^+^), with some dehydration modifications ([M + H – H_2_O]^+^). The collision energy ranged from 15 to 40 eV, and MF and RMF scores confirmed high spectral matching accuracy for all identified oligosaccharides.

### 3.7 Blood and blood serum analysis

Blood serum analysis was conducted to assess the biochemical markers of the experimental groups (Table 4). Total protein and albumin levels were significantly different between groups, with LDM-2 exhibiting the highest total protein (83.10 ± 0.35 g/L, *p* ≤ 0.05) and a lower albumin concentration (30.30 ± 0.56 g/L, *p* ≤ 0.05) compared to the control (GC-1), and HDM3 groups. Albumin/Globulin (A/G) ratio showed a marked decrease in the LDM-2 group (0.65 ± 0.34, *p* ≤ 0.05), suggesting potential alterations in protein metabolism. Urea and creatinine levels were also elevated in the LDM-2 and HDM-3 groups, with creatinine in HDM-3 (47.00 ± 0.18 μmol/L) significantly higher than in GC-1 (37.10 ± 0.33 μmol/L, *p* ≤ 0.05), indicating possible kidney function modulation. ALT and AST enzyme levels showed moderate differences, with AST levels increasing in the LDM-2 group. Alkaline phosphatase (ALP) activity was significantly higher in the LDM-2 group (150.01 ± 0.33 U/L, *p* ≤ 0.05). Glucose levels varied, with the HDM-3 group showing an increase (5.02 ± 0.27 mmol/L, *p* ≤ 0.05), while LDM-2 exhibited the lowest glucose concentration (2.98 ± 0.17 mmol/L). These findings suggest that mare's milk supplementation influences key biochemical parameters, particularly protein metabolism, enzyme activity, and glucose regulation.

**Table 4:** Blood serum analysis.

**Indicator**	**GC-1**	**LDM-2**	**HDM-3**	***p-value***
Total protein, g/L	73.00 ± 0.27^a^	83.10 ± 0.35^b^	79.20 ± 0.92^ab^	≤0.05
Albumin, g/L	36.60 ± 0.38^a^	30.30 ± 0.56^b^	37.60 ± 0.56^a^	≤0.05
Albumin/Globulin Ratio (A/G)	0.91 ± 0.25^a^	0.65 ± 0.34^b^	0.90 ± 0.45^a^	≤0.05
Urea, mmol/L	5.60 ± 0.14^a^	6.80 ± 0.27^b^	5.90 ± 0.28^ab^	0.06
Creatinine, μmol/L	37.10 ± 0.33^a^	43.70 ± 0.28^b^	47.00 ± 0.18^b^	≤0.05
ALT, U/L	38.01 ± 0.23^a^	40.01 ± 0.36^ab^	38.10 ± 0.25^a^	0.07
AST, U/L	130.10 ± 0.37^a^	140.15 ± 0.29^ab^	136.50 ± 0.16^ab^	0.08
ALP, U/L	120.05 ± 0.29^a^	150.01 ± 0.33^b^	123.02 ± 0.29^ab^	≤0.05
GGT, U/L	2.70 ± 0.35^a^	2.30 ± 0.25^a^	2.40 ± 0.45^ab^	0.09
Glucose, mmol/L	4.20 ± 0.21^a^	2.98 ± 0.17^b^	5.02 ± 0.27^ab^	≤0.05

Values are expressed as mean ± SD, and statistical significance was evaluated. When p ≤ 0.05, Tukey's *post hoc* test was used for multiple comparisons. Different superscript letters (a, b) indicate statistically significant differences (p ≤ 0.05) between groups. Groups sharing the same letter are not significantly different.

The hematological analysis revealed significant changes in WBC count, lymphocytes, monocytes, and granulocytes across the experimental groups (Table 5). The LDM-2 group exhibited the highest WBC count (19.31 ± 0.17 × 10^9^/L, *p* ≤ 0.05), whereas the GC-1 group had the lowest (8.20 ± 0.23 × 10^9^/L). Lymphocyte and monocyte counts were also significantly increased in the LDM-2 group, suggesting an immune response to mare's milk intake. Granulocyte levels were significantly elevated in the LDM-2 group (5.71 ± 0.45 × 10^9^/L, *p* ≤ 0.05) compared to the GC-1 and HDM-3 groups.

**Table 5:** Hematological analysis.

**No**.	**Parameters**	**GC-1**	**LDM-2**	**HDM-3**
**Leicocytes**				
1	WBC × 10^9^/L	8.20 ± 0.23^a^	19.31 ± 0.17^b^	11.20 ± 0.05^ab^
2	Lymphocytes × 10^9^/L	1.81 ± 0.44^a^	5.9 ± 0.22^b^	1.73 ± 0.34^b^
3	Monocytes × 10^9^/L	0.90 ± 0.25^a^	1.52 ± 0.12^ab^	1.02 ± 0.06^ab^
4	Granulocytes × 10^9^/L	1.12 ± 0.27^a^	5.71 ± 0.45^b^	2.72 ± 0.37^b^
5	Lymphocytes, %	84.11 ± 0.68^a^	90.33 ± 1.6^b^	85.00 ± 0.27^ab^
6	Monocytes, %	8.23 ± 0.26^a^	14.31 ± 0.23^b^	9.90 ± 0.57^bc^
7	Granulocytes, %	32.11 ± 1.25^a^	40.90 ± 0.3^b^	32.11 ± 0.33^fd^
**Eritrocites**				
1	RBC x 10^12^/L	6.51 ± 0.48^a^	9.86 ± 0.13^b^	7.15 ± 0.39^ab^
2	Hb, g/L	164.34 ± 0.25^a^	164.17 ± 0.37^b^	155.57 ± 0.17^bc^
3	HCT, %	40.21 ± 0.48^a^	49.15 ± 0.12^b^	45.23 ± 0.78^bc^
4	MCV, fL	53.51 ± 0.33^a^	62.37 ± 0.63^b^	52 ± 0.87^bc^
5	MCH, pg	16.52 ± 0.26^a^	16.63 ± 0.55^b^	16.73 ± 0.11^b^
6	MCHC, g/L	309.12 ± 0.87^a^	360.69 ± 0.19^b^	334.37 ± 0.96^bc^
7	RDW, %	13.92 ± 0.12^a^	15.77 ± 0.27^b^	14.72 ± 0.19^b^
8	PLT x 10^9^/L	680.07 ± 0.12^a^	833.27 ± 0.05^b^	580.95 ± 0.88^bc^

Values are presented as Mean ± standard deviation with n = 6. Values with different superscript along the same row are significantly different at p < 0.05. Here, WBC, White Blood Cells, RBC, Red Blood Cells, Hb, Hemoglobin, HCT, Hematocrit, MCV, Mean Corpuscular Volume, MCH, Mean Corpuscular Hemoglobin, MCHC, mean corpuscular hemoglobin concentration, RDW, Red Cell Distribution Width, PLT, Platelet.

In the erythrocyte parameters, the LDM-2 group showed increased RBC count (9.86 ± 0.13 × 10^12^/L, *p* ≤ 0.05) and hematocrit levels (49.15 ± 0.12%, *p* ≤ 0.05) compared to the control. However, hemoglobin levels remained similar between GC-1 and LDM-2 but were slightly lower in the HDM-3 group. The MCV was significantly higher in LDM-2 (62.37 ± 0.64 fL, *p* ≤ 0.05), indicating a shift in RBC size. PLT also varied, with a significant increase in LDM2 (833.27 ± 0.05 × 10^9^/L, *p* ≤ 0.05), while the HDM-3 group exhibited a moderate decrease (580.95 ± 0.88 × 10^9^/L).

### 3.8 Histomorphological analysis

**Figure 4:**
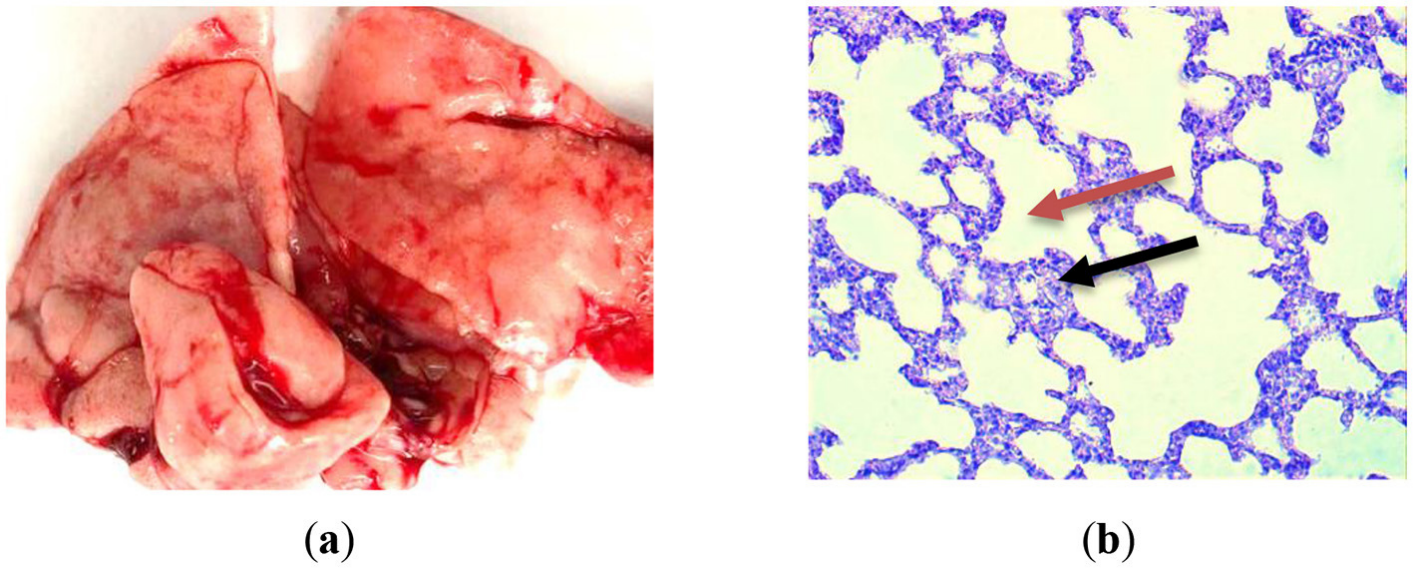
Lung histological analysis of group GC-1. **(a)** The illustration of normal lung; **(b)** Hematoxylin-eosin staining. Magnification is ×400. The red arrow is alveoli; the black one is interalveolar partition.

Histomorphological changes in the lungs of control animals from the first group (GC-1), which consumed mare's milk powder for duration of 30 days, were examined. Histological examination of lung tissue samples from group GC-1 revealed histological features consistent with a normal, healthy state. These observations were substantiated through a thorough analysis of the specimens. The lung tissue exhibited a well-preserved alveolar architecture. Alveoli, the primary sites of gas exchange in the lung, appeared intact and appropriately inflated, indicating the absence of pathological changes (Figures 4a, b). The bronchial tree demonstrated normal bronchioles, bronchi, and their associated structures. The bronchioles showed a typical layering of smooth muscle cells and an intact epithelial lining, with no signs of inflammation, fibrosis, or excessive mucus production. The connective tissue framework, particularly the collagen fibers within the interstitium, displayed a regular and supportive network, which is essential for maintaining the lung's structural integrity. The respiratory epithelium lining the alveoli and airways appeared undamaged and exhibited a characteristic simple squamous epithelium for efficient gas exchange. There were no signs of hyperplasia or metaplasia. The lung tissue is pink in color without signs of edema or hemorrhage. The cut surface of the lung is smooth, soft, and rich pink in color, with moderate blood filling (Figure 4a). Morphological studies of the lungs of rats of the first group on semi-thin sections showed that the pulmonary pleura was preserved, and the intrapulmonary airways and parenchyma were unchanged, consisting of the airways and the respiratory section having a characteristic structure. The walls of the medium-sized bronchi are covered with single-layer multirow ciliated epithelium. Rare small-focal infiltration of alveolar walls with lymphocytes was noted (Figure 4b).

Therefore, a histological examination of the lungs in the initial control group of rats revealed their normalcy, with the preservation of their structural integrity. No distinct pathological or physiological alterations were detected.

After being infected with *S. pneumoniae* 79 strains (0.3 ml of a 1,000 CFU mixture), the second group of animals (LDM-2) was supplemented with 0.1 g of low-dose dry mare's milk for duration of 30 days. Histomorphological alterations were observed in the lungs of animals belonging to the LDM-2.

**Figure 5:**
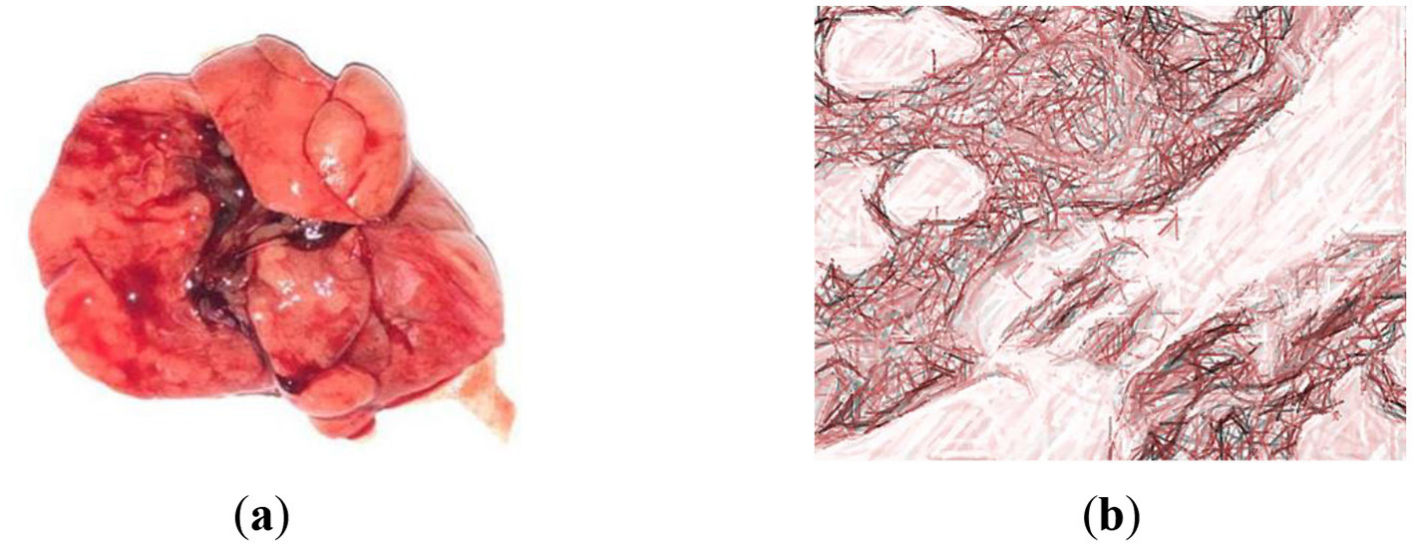
Examination of macro and microscopic specimens of the lungs from rats infected with strains of *S. pneumoniae* 79. **(a)** The lung of Wistar rat from group LDM-2; **(b)** Hematoxylin-eosin staining. Magnification is ×400. Thinned interalveolar septa are noticed.

When studying macro preparations of the lungs of rats against the background of the administration of LDM-2, it is clear that the lungs increased in size, and their anterior edges overlap each other (Figure 5a). The lung tissue exhibited signs of acute inflammation characterized by the presence of inflammatory cells, such as neutrophils and macrophages, within the alveoli and interstitial spaces. This suggests a host response to infection (Figure 5b). Moreover, several alveoli showed signs of structural damage, including thickening of the alveolar walls and the disruption of the normal architecture. This can result in impaired gas exchange. Additionally, bronchioles and bronchi displayed alterations, including epithelial sloughing and increased mucus production, indicative of airway inflammation. The lung tissue is highly airy and light.

These histomorphological alterations are indicative of a response to the infection with *S. pneumoniae* 79 strains and provide insights into the pathological changes in the lungs of animals in the LDM-2 group.

**Figure 6:**
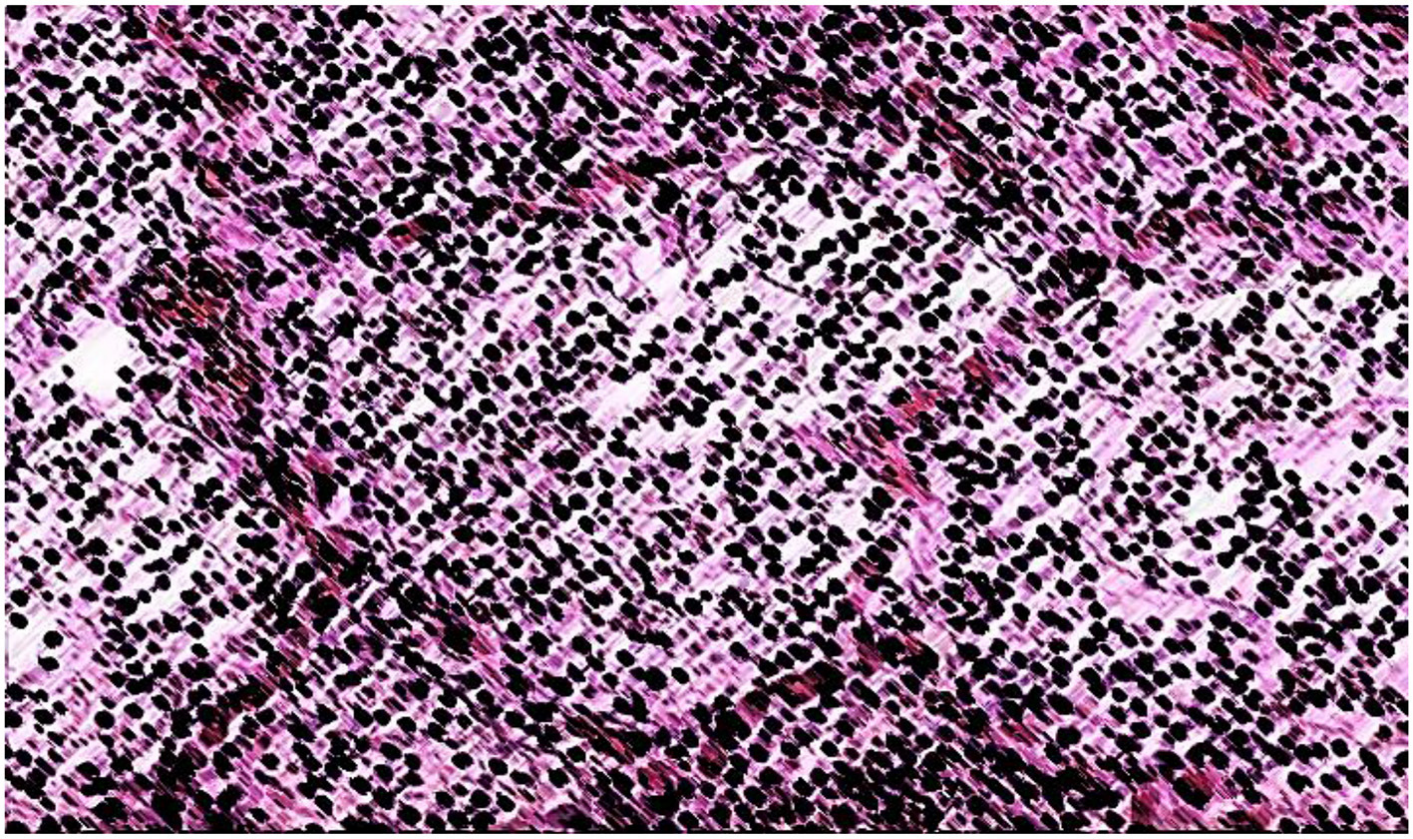
Croupous pneumonia, gray hepatic stage (in the context of croupous pneumonia, the “gray hepatic stage” suggests a specific stage of the disease characterized by the appearance of grayish or hepatictissue due to inflammatory changes). Hematoxylin-eosin staining. Magnification is ×400.

The morphological examination of lung tissue, as shown in Figure 6, revealed significant pathological changes in the second group. Observations included the widening of respiratory bronchioles and alveolar lumens, disruption and tearing of interalveolar septa, and thickening with sclerotic changes in vessel walls. Lung tissue exhibited increased vascularity, fibrin accumulation, and neutrophils within alveolar lumens, with macrophages engaging in bacterial phagocytosis. Hemolysis of red blood cells was noted. Structural integrity loss was evident, with septal destruction impairing gas exchange. Pulmonary vessel thickening suggested vascular remodeling. The lung appeared congested, likely due to inflammatory responses to *S. pneumoniae* infection.

In Wistar rats, infection with *S. pneumoniae* 79 led to a variety of symptoms and clinical manifestations. The specific symptoms can vary depending on the severity of the infection, the strain of *S. pneumoniae* 79, and the host's immune response. In our case, common symptoms and signs were observed after infection with *S. pneumoniae* in Wistar rats include respiratory distress, lethargy, nasal discharge, weight loss, and decreased activity. Rats exhibited signs of respiratory distress, such as rapid and labored breathing, coughing, and wheezing. These symptoms reflect the lung's involvement in the infection. Infected rats appeared lethargic and less active than usual. They spent more time resting and had a decreased interest in normal activities. Additionally, rats had a nasal discharge, which was clear or purulent, as a result of possible upper respiratory tract involvement. Moreover, rats huddled together for warmth and comfort.

**Figure 7:**
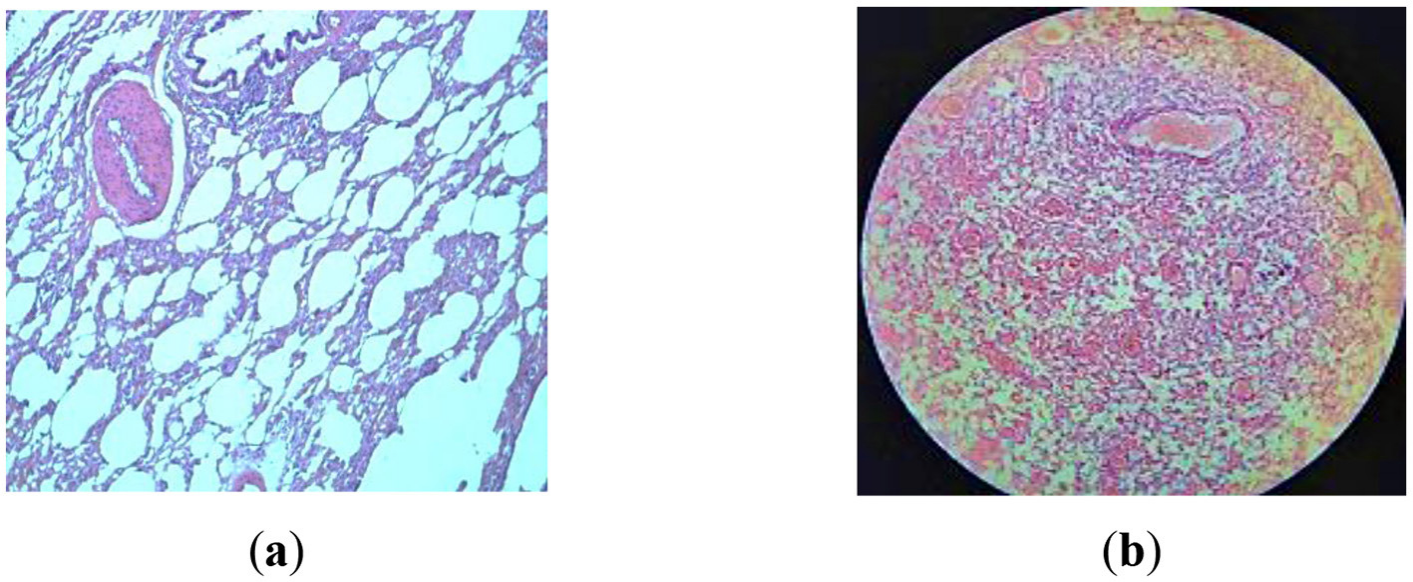
Examination of macro and microscopic specimens of the lungs from HDM-3 rats infected with strains of *S. pneumoniae* 79 after being treated with 1.5 g of mare's milk over 30 days. Hematoxylin-eosin staining. **(a)** Magnification is ×400; **(b)** Magnification is ×200.

The histological analysis of lung tissues from the third group of rats HDM-3 reveals a predominant presence of alveoli filled with a uniform pale pink substance (Figure 7). Only a minority of alveoli shows an absence of exudate, but their lumens remain unexpanded, with a diameter approximately equivalent to that of 2–3 red blood cells. Consequently, these areas exhibit a nodular thickening, protruding into the capillary lumens. In regions where the alveoli are engorged with exudate, red blood cells are displaced from the capillaries, leading to the depletion of blood within these capillaries. Small arteries and veins display relatively minor dilation and partial blood filling. Additionally, we observed compensatory-adaptive responses that exhibited physiological and biochemical properties aimed at enhancing the rats' adaptive capacities. In this context, it is conceivable that the administration of high-dose dry mare's milk (1.5 g) may serve to prevent or mitigate the severity of toxic pulmonary edema, preserving the integrity of alveolar lumens. Moreover, we noted the presence of perivascular and peribronchial infiltration, along with the proliferation of soft fibrous connective tissue. Notably, the lungs of rats in the HDM-3 group closely resembled those of the GC-1 group, displaying no discernible changes in size or appearance when compared to the lungs of rats from the LDM-2 group.

Histomorphological analysis revealed notable distinctions between the LDM-2 and HDM-3 groups. The HDM-3 group exhibited reduced pathological changes, with no distinct pathological or physiological abnormalities. Signs of healing in *S. pneumoniae*-infected HDM-3 rats included improved breathing, diminished coughing and wheezing, and increased activity. Appetite recovery led to weight gain after the second week. Nasal discharge, present for two weeks, decreased and was fully resolved by the fourth week.

## 4 Discussion

During a 6-month lactation period, the physicochemical properties of Adaev horse milk exhibited variations, providing insights into its composition and distinguishing it from other horse breeds (Table 1). The total solids content averaged 11.02%, with the lowest (10.07%) in the first month and the highest (12.15%) in the fifth month. Milk fat, the most variable component, had a coefficient of variation of 59%, ranging from 0.87% to 2.97%, aligning with reported values of 0.8–2.2% during lactation (35, 36). The protein content averaged 1.76%, higher than in Wielkopolski, Polish konik, and Polish coldblood breeds (1.3%), reaching its peak (2.35%) in the fourth month (37–43). Moreover, the physical properties of mare's milk fluctuated due to factors such as diet and lactation stage. Titratable acidity ranged from 1.25 to 3.49 °SH, decreasing similarly to lactose concentration over six months, although no direct correlation was established (Table 6). The pH value remained relatively stable between 6.87 and 7.07, slightly more alkaline than cow's milk (pH ~6.6) (44–49). The freezing point, influenced by lactose content, varied from −0.5356 to −0.5476°C, lower than that of cow's milk (−0.5 to −0.55°C) (45).

**Table 6:** Physical properties of mare's milk.

**Month**	**Titratable acidity, °SH**	**pH-value**	**Freezing point, °C**
1st	3.49 ± 0.12^*^	6.87 ± 0.08	−0.5453 ± 0.005
2nd	3.37 ± 0.11^*^	6.91 ± 0.09	−0.5476 ± 0.004
3rd	2.63 ± 0.15	6.93 ± 0.07	−0.5465 ± 0.006
4th	2.48 ± 0.14	7.07 ± 0.05^*^	−0.5454 ± 0.005
5th	2.35 ± 0.13	7.01 ± 0.06	−0.5456 ± 0.004
6th	1.25 ± 0.10^*^	6.99 ± 0.05	−0.5356 ± 0.007^*^
Mean ± SD	2.59 ± 0.13	6.96 ± 0.07	−0.5443 ± 0.006
*p*-value	≤0.05	0.03	≤0.05

* indicates statistically significant differences (p ≤ 0.05).

Lyophilized mare's milk, widely used as a nutritional supplement, retained its key components in powder form, including fat (0.62–0.91%), protein (2.0–2.85%), lactose (7.26–9.97%), casein (1.61–2.20%), lactic acid (0.089–0.076%), and citric acid (0.14–0.2%) (Table 2). Among these components, casein, a key protein influencing milk coagulation, ranged from 0.13% to 1.08%, decreasing progressively over lactation. This aligns with previous studies indicating that mare's milk is albumin-rich, with casein constituting less than 50% of total proteins (40). The variability in casein content is likely influenced by dilution effects, hormonal changes, and foal diet transitions (41, 42). Compared to cow's milk, Adaev horse milk contains less casein but a higher proportion of whey proteins, enhancing digestibility and bioavailability. SDS PAGE analysis confirmed this protein composition, revealing dominant bands at ~40 kDa, ~25 kDa, and ~15 kDa, corresponding to β-lactoglobulin, α-lactalbumin, and serum albumin, respectively (Figure 3). The presence of lower molecular weight peptides (~5–15 kDa) suggests potential bioactive functions, which may contribute to the nutritional and functional properties of mare's milk. Additionally, lactose, the primary carbohydrate, ranged from 6 to 7%, contributing to milk sweetness and serving as an energy source for foals. Adaev horse milk had average lactose content of 6.26%, which is higher than that of cows, goats, and sheep's milk (43). Furthermore, HILIC-MS analysis identified key sialylated oligosaccharides, including 3′SL, 6′SL, 3′SLN, LSTa, LSTb, and LSTc, which are known for their prebiotic and immunomodulatory properties.

Oligosaccharides play a crucial role in modulating gut microbiota, supporting immune function, and enhancing gut barrier integrity (7). Compared to plant-derived dietary fibers and oligosaccharides in standard chow feed, which promote gut health primarily through fermentation and short-chain fatty acid production, mare's milk oligosaccharides resemble those found in human milk, potentially exerting unique prebiotic and immunomodulatory effects (6, 7). Specifically, sialylated oligosaccharides (e.g., 3′SL, 6′SL, and 3′SLN) have been known to selectively promote the growth of beneficial gut bacteria such as Bifidobacterium and Lactobacillus, contributing to microbiome diversity and improved immune function (17, 31). Given their prebiotic potential, the effects of mare's milk oligosaccharides on gut microbiota composition and systemic immune responses warrant further investigation.

The microbiota present in mare's milk comprises a heterogeneous assemblage of advantageous bacterial species, such as Lactobacillus and Bifidobacterium, which are integral to the modulation of the immune system and the promotion of gastrointestinal health (7, 31). These probiotic microorganisms facilitate immune enhancement by fostering the maturation of gut-associated lymphoid tissue and stimulating the synthesis of anti-inflammatory cytokines, such as IL-10 (33). Moreover, they contribute to the equilibrium between pro-inflammatory and regulatory immune responses, an aspect that is particularly critical for the immunological development of neonates (49). The microbiota of mare's milk also demonstrates antimicrobial properties through the production of bacteriocins and organic acids, which serve to inhibit the proliferation of pathogenic bacteria within the gastrointestinal tract. Additionally, their interaction with oligosaccharides found in mare's milk augments their prebiotic effects, thereby promoting the proliferation of beneficial microbes while simultaneously modulating intestinal permeability and immune tolerance (50, 51). Collectively, these microbiota-driven processes play a significant role in fortifying the immune system, alleviating inflammation, and fostering the establishment of a robust gut microbiome in the host.

In this study, compared to the unsupplemented control group (GC-1), rats receiving mare's milk supplementation (LDM-2 and HDM-3) exhibited notable changes in blood serum protein levels, indicating a potential impact on protein metabolism and immune modulation. SDS-PAGE analysis confirmed that Adaev horse milk has a high whey protein content and lower casein concentration, contributing to enhanced digestibility and bioavailability of essential amino acids. These differences in protein composition may explain the elevated serum protein levels and potential immune responses observed in supplemented groups. Furthermore, histological examinations revealed that high-dose supplementation (HDM-3) influenced tissue morphology, particularly in immune-related organs. This observation aligns with the hypothesis that mare's milk supplementation may modulate inflammatory responses and enhance tissue recovery following bacterial infection. The relationship between Adaev horse milk composition, dietary supplementation, and changes in feed due to supplementation is further supported by blood serum analysis and histological findings, indicating potential systemic benefits beyond standard chow feed alone.

The standard rat chow used in the study contained 4.5% fat, 0.02% cholesterol, ~19.7% protein, 4.3% dietary fiber, 51% sugar, and provided 3.2 kcal/g. The daily chow intake per rat was estimated at 20.52 g/day, resulting in a total caloric intake of ~64 kcal/day for the control group (GC-1). The LDM-2 group received chow supplemented with 0.1 g of lyophilized mare's milk powder, contributing an additional 0.4 kcal/day, 0.002–0.0025 g protein, and 0.045 g sugar (mainly lactose), with negligible dietary fiber. This brought their total estimated daily caloric intake to 64.4 kcal/day, protein intake to 3.6–4.0 g/day, and sugar intake to ~0.645 g/day. The HDM-3 group received 1.5 g of lyophilized mare's milk powder, adding ~6 kcal/day, 0.03–0.0375 g protein, and 0.675 g sugar, increasing their total daily caloric intake to ~70 kcal/day, protein intake to 3.63–4.04 g/day, and sugar intake to ~1.275 g/day.

Given that weight gain is primarily influenced by caloric intake, the additional 6 kcal/day in the HDM-3 group likely contributed to the observed differences. However, other factors, such as the presence of bioactive compounds in mare's milk, may have also played a role in metabolic regulation and nutrient utilization. Bioactive peptides in dairy products have been shown to exert a variety of physiological effects, including antihypertensive, antimicrobial, immunomodulatory, and opioid-like properties (52). These bioactive compounds can influence metabolism by interacting with the endocrine system, modulating gut microbiota composition, and affecting insulin sensitivity, which could contribute to the observed differences in weight gain. The higher protein intake in the HDM-3 group may have influenced satiety and metabolic rate. Protein consumption has been well-documented to increase diet-induced thermogenesis, leading to higher energy expenditure compared to carbohydrates and fats (53). Additionally, protein enhances satiety by stimulating the release of appetite-regulating hormones, such as glucagon-like peptide-1 and peptide YY, while simultaneously reducing levels of ghrelin, the hunger hormone (54). These mechanisms likely contributed to differences in metabolic responses among the groups. Variations in dietary fiber intake could have affected nutrient absorption and gut microbiota composition. Dietary fiber is known to slow gastric emptying, reducing the rate of glucose absorption and thereby improving metabolic control (55). Additionally, fermentable fibers, such as those found in some components of mare's milk, serve as a substrate for gut microbiota, promoting the production of short-chain fatty acids, which have been linked to improved insulin sensitivity and lipid metabolism (56). Furthermore, the increased sugar content in the HDM-3 group may have impacted energy availability and insulin response, further influencing overall metabolic outcomes. Studies have demonstrated that high sugar intake, particularly from rapidly digestible sugars such as lactose, can lead to postprandial hyperglycemia, which in turn stimulates insulin secretion and fat storage (52, 57).

A blood serum analysis was conducted in Wistar rats following *S. pneumoniae* infection to assess health and physiological changes (Table 4). Total protein levels, which typically range from 5.5 to 7.5 g/dL in healthy Wistar rats (50), were elevated in the supplemented groups, with mean concentrations of 83.10 ± 0.35 g/L in LDM-2 and 79.20 ± 0.92 g/L in HDM-3, compared to 73.00 ± 0.27 g/L in the control group (GC-1). This increase is likely due to immune activation, as proinflammatory cytokines and acute-phase proteins contribute to fluctuations in serum protein levels, which vary based on infection severity, immune response, and pathogen virulence (50).

Albumin levels, typically between 35 and 55 g/L (51), were lower in LDM-2 (30.30 ± 0.56 g/L) but remained within normal limits in GC-1 (36.60 ± 0.38 g/L) and HDM-3 (37.60 ± 0.56 g/L). This decline in LDM-2 may be attributed to infection-induced liver dysfunction and an increased production of acute-phase proteins, which suppress hepatic albumin synthesis. Similarly, the albumin/globulin (A/G) ratio, an indicator of immune balance, ranged from 0.91 ± 0.25 in GC-1 to 0.65 ± 0.35 in LDM-2 and 0.90 ± 0.45 in HDM-3, with the lower A/G ratio in LDM-2 likely due to elevated globulin levels from acute-phase protein production, including C-reactive protein and alpha-1-acid glycoprotein (58).

Kidney function markers, such as blood urea (10–25 mg/dL; 3.57–8.93 mmol/L) and creatinine (0.2–1.5 mg/dL; 17.7–133 μmol/L) (58), showed slight variations between groups but remained within normal physiological ranges. Urea levels were 5.60 ± 0.14 mmol/L in GC-1, 6.80 ± 0.27 mmol/L in LDM-2, and 5.90 ± 0.28 mmol/L in HDM-3. Creatinine levels followed a similar trend, increasing from 37.10 ± 0.33 μmol/L in GC-1 to 43.70 ± 0.28 μmol/L in LDM-2 and 47.00 ± 0.18 μmol/L in HDM-3. These results suggest that mare's milk supplementation did not induce significant renal impairment.

Similarly, ALT, AST, ALP, and GGT enzyme levels, typically associated with liver dysfunction, showed no abnormalities, suggesting that mare's milk supplementation did not adversely affect liver function. ALT levels were 38.01 ± 0.23 U/L in GC-1, 40.01 ± 0.36 U/L in LDM-2, and 38.10 ± 0.25 U/L in HDM-3. AST levels followed a similar pattern, with 130.10 ± 0.37 U/L in GC-1, 140.15 ± 0.29 U/L in LDM-2, and 136.50 ± 0.35 U/L in HDM-3. ALP levels were significantly higher in LDM-2 (150.01 ± 0.31 U/L) compared to GC-1 (120.05 ± 0.29 U/L) and HDM-3 (123.02 ± 0.28 U/L), suggesting a potential effect on bone metabolism or hepatic function. GGT levels remained stable across groups, with 2.70 ± 0.35 U/L in GC-1, 2.36 ± 0.45 U/L in LDM-2, and 2.40 ± 0.58 U/L in HDM-3.

A hematological analysis was conducted in Wistar rats following *S. pneumoniae* infection to assess changes in blood parameters and immune response (Table 5). CBC analysis provides critical information about WBCs, RBCs, Hb, and other hematological indices, helping to evaluate the severity of infection and immune activation. Leukocytosis, characterized by an elevated WBC count, is a common response to bacterial infections, reflecting the mobilization of immune defenses. The typical WBC range in healthy Wistar rats is 6,000 to 12,000 cells/μL, but during infection, WBC counts often increase substantially, depending on the infection stage, severity, and host immune response (59). Differential WBC analysis, which includes neutrophils, lymphocytes, monocytes, eosinophils, and basophils, provides insights into immune activation patterns and the progression of infection (60). These parameters are crucial for determining immune function, inflammatory response, and the effectiveness of host defense mechanisms against *S. pneumoniae*.

In healthy Wistar rats, the typical total WBC count ranges from 6 to 10 × 10^9^ cells/L (61). Following *S. pneumoniae* infection, a significant increase in WBC count (leukocytosis) is expected as the immune system mobilizes its defenses. In this study, LDM-2 rats exhibited a markedly elevated WBC count of 19.31 × 10^9^ cells/L, indicating a strong immune response to infection. In contrast, HDM-3 rats showed a more moderate WBC increase (11.20 × 10^9^ cells/L), suggesting that the infection had progressed beyond the initial immune activation phase. The degree of WBC elevation depends on infection severity, stage, and individual immune response, with higher counts reflecting active inflammation and pathogen clearance (62). These findings highlight the dose-dependent impact of mare's milk supplementation on immune function, potentially modulating infection-induced hematological responses.

During infection progression, particularly in the later immune response stages, an increase in lymphocytosis is commonly observed, reflecting adaptive immune activation. In healthy Wistar rats, lymphocyte counts typically range from 1.5 to 6 × 10^9^ cells/L (63). In this study, lymphocyte levels remained stable across most groups, except for LDM-2 rats, which exhibited elevated counts of 5.9 × 10^9^ cells/L, indicating a stronger immune response to infection. Additionally, monocyte and granulocyte levels in LDM-2 rats exceeded normal reference values (0.1–1.0 × 10^9^ cells/L for monocytes and 1.0–3.5 × 10^9^ cells/L for granulocytes), reaching 1.52 × 10^9^ cells/L and 5.71 × 10^9^ cells/L, respectively. This aligns with findings from other studies indicating that systemic inflammation enhances the biological response to mobilize additional cells from the central and peripheral immune/hematopoietic systems (64). Additionally, inflammation is associated with changes in cytokine and chemokine profiles, which differentially affect myelopoiesis and cellular mobilization (65). Furthermore, the activation of pattern recognition receptors by damage-associated molecular patterns and pathogen-associated molecular patterns can induce the production of pro-inflammatory cytokines and promote immune cell localization to sites of injury (66). Moreover, RBC parameters in LDM-2 rats were elevated, with RBC counts of 9.86 × 10^12^ cells/L, mean corpuscular volume of 62.37 fL, and mean corpuscular hemoglobin concentration of 360.69 g/L, surpassing normal reference values. The observed hematological alterations in LDM-2 rats suggest a heightened immune response and potential alterations in erythropoiesis due to infection or inflammation-related metabolic shifts, as inflammatory cytokines such as IL-6 and IFN-γ can suppress erythropoiesis during infection (67).

Histological analysis of lung tissues in Wistar rats revealed significant differences among the experimental groups. The control group (GC-1) exhibited normal lung histology, with intact alveolar structures, bronchial elements, pulmonary blood vessels, and respiratory epithelium, indicating no inflammation or pathological changes, establishing a baseline for healthy Wistar rats. In contrast, the LDM-2 group showed marked histopathological changes due to pneumonia, including inflammatory cell infiltration, alveolar damage, and structural disorganization, reflecting severe lung injury. In the HDM-3 group, less pronounced structural damage and reduced inflammatory and dystrophic processes were observed, suggesting a protective effect of mare's milk in mitigating pneumonia-associated lung damage. This observation aligns with findings from other studies that have demonstrated the health benefits of mare's milk. For instance, research has shown that fermented mare's milk possesses anti-inflammatory properties, potentially beneficial for relieving inflammatory diseases (68). By day 30, the HDM-3 group exhibited enhanced compensatory-adaptive reactions and improved lung tissue integrity, highlighting the potential therapeutic benefits of mare's milk in lung repair. These findings suggest that mare's milk supplementation may help reduce toxic accumulation, enhance non-specific immune resistance, and provide essential vitamins and microelements that counteract respiratory disease severity, ultimately contributing to reduced pulmonary complications and improved recovery from bronchopneumonia (65–68).

Thus, mare's milk powder is an important source of high-quality nutritional content and biological value, superior in its dietary and medicinal properties. It gives the body vigor and strength, has a beneficial effect on the nervous system, normalizes metabolism, the secretory activity of the digestive organs, has bactericidal properties, and the ability to secrete antibiotic substances that prevent and treat tuberculosis, cardiovascular diseases, the excretory and digestive systems. Mare's milk as a dietary supplement significantly affects body weight loss by regulating lipid levels. Metabolism in a rat model of induced pneumonia showed that mare's milk strengthens the immune system, changing the composition and structure of the intestinal microflora due to its antiviral, antibacterial, and immunostimulating properties. It promotes the immune function of the immune system and improves the immune response in rats with pneumonia. In addition, it can act as an effective dietary supplement for the prevention and treatment of pneumonia or tuberculosis, as well as diseases associated with pneumonia.

## 5 Conclusions

In conclusion, mare's milk, particularly from the Adaev horse breed, exhibits a unique biochemical composition that supports its immunomodulatory potential, making it a promising functional food and therapeutic agent. Its rich content of bioactive proteins, oligosaccharides (e.g., 3′SL, 6′SL, and 3′SLN), and essential nutrients, combined with its similarity to human milk, highlights its potential role in immune support and disease prevention. Furthermore, the high whey protein content and lower casein levels in mare's milk enhance digestibility and the bioavailability of essential amino acids, contributing to its anti-inflammatory effects.

This is supported by the findings of this study, which demonstrated the immunomodulatory effects of mare's milk supplementation. Rats in the LDM-2 group exhibited significant inflammatory and structural lung damage, including alveolar interstitial edema, venous and capillary congestion, and infiltration of fibrous connective tissue, indicating a heightened inflammatory response to infection. In contrast, HDM-3 supplementation resulted in fewer pathological changes, with only minor, fully reversible compensatory-adaptive modifications, suggesting that higher doses of mare's milk exert a protective effect on lung tissue.

Additionally, these findings confirm that mare's milk enhances immune resilience through its bioactive components, particularly oligosaccharides and proteins, which collectively modulate inflammation, support gut-immune axis interactions, and aid in tissue recovery. Given its promising immunomodulatory properties, mare's milk holds significant potential as a natural therapeutic agent, warranting further research into its broader clinical applications and long-term health benefits.

## Data Availability

The raw data supporting the conclusions of this article will be made available by the authors, without undue reservation.

## References

[1] Aimen A Moldasheva A Dossymova O Atasheva D Aimenova S. Current status of dairy products in the republic of Kazakhstan. Open J Bus Manage. (2022) 10:2432–41. 10.4236/ojbm.2022.105122

[2] Górska-Warsewicz H Rejman K Laskowski W Czeczotko M. Milk and dairy products and their nutritional contribution to the average Polish diet. Nutrients. (2019) 11:1771. 10.3390/nu11081771 31374893 PMC6723869

[3] Vedovato GM Vilela S Severo M Rodrigues S Lopes C Oliveira A. Ultra-processed food consumption, appetitive traits, and BMI in children: a prospective study. Br J Nutr. (2021) 125:1427–36. 10.1017/S0007114520003712 32962770

[4] Garvey SM Mah E Blonquist TM Kaden VN Spears JL. The probiotic *Bacillus subtilis* BS50 decreases gastrointestinal symptoms in healthy adults: a randomized, double-blind, placebo-controlled trial. Gut Microbes. (2022) 14:2122668. 10.1080/19490976.2022.2122668 36269141 PMC9590435

[5] Koilybayeva M Shynykul Z Ustenova G Abzaliyeva S Alimzhanova M Amirkhanova A. Molecular characterization of some *Bacillus* species from vegetables and evaluation of their antimicrobial and antibiotic potency. Molecules. (2023) 28:3210. 10.3390/molecules28073210 37049972 PMC10095821

[6] Musaev A Sadykova S Anambayeva A Saizhanova M Balkanay G Kolbayev M. Mare's milk: composition, properties, and application in medicine. Arch Razi Inst. (2021) 76:1125–35. 10.22092/ari.2021.355834.1725 35096348 PMC8790991

[7] Pietrzak-Fiećko R Kamelska-Sadowska AM. The comparison of nutritional value of human milk with other mammals' milk. Nutrients. (2020) 12:1404. 10.3390/nu12051404 32422857 PMC7284997

[8] Debashree R Aiqian Y Moughan PM Singh H. Composition, structure, and digestive dynamics of milk from different species—a review. Front Nutr. (2020) 7:577759. 10.3389/fnut.2020.577759 33123547 PMC7573072

[9] Markiewicz-Keszycka M Wójtowski J Czyżak-Runowska G Kuczyńska B Puppel K Krzyżewski J. Concentration of selected fatty acids, fat-soluble vitamins, and β-carotene in late lactation mares' milk. Int Dairy J. (2014) 38:31–6. 10.1016/j.idairyj.2014.04.003

[10] Faccia M D'Alessandro AG Summer A Hailu Y. Milk products from minor dairy species: a review. Animals. (2020) 10:1260. 10.3390/ani10081260 32722331 PMC7460022

[11] Barłowska J Polak G Janczarek I Tkaczyk E. The influence of selected factors on the nutritional value of the milk of cold-blooded mares: the example of the Sokólski breed. Animals. (2023) 13:1152. 10.3390/ani13071152 37048410 PMC10093385

[12] Holmes AD Spelman AF Smith CT Kuzmeski JW. Composition of mares' milk as compared with that of other species. J Dairy Sci. (1947) 30:385–95. 10.3168/jds.S0022-0302(47)92363-1

[13] Polish Committee for Standardization. Milk—Determination of nitrogen content—Part 3: Block-digestion method (semi-micro rapid routine method). Warsaw, Poland: Polish Committee for Standardization (2008).

[14] Kazimierska K Kalinowska-Lis U. Milk proteins—their biological activities and use in cosmetics and dermatology. Molecules. (2021) 26:3253. 10.3390/molecules26113253 34071375 PMC8197926

[15] Park YW editor. Bioactive Components in Milk and Dairy Products. Ames, IA: Wiley-Blackwell (2009).

[16] Reiter AS Sarah AR. Lactation in horses. Anim Front. (2023) 13:96–100. 10.1093/af/vfad003 37324210 PMC10266743

[17] Walstra P Wouters JTM Geurts TJ. Dairy Science and Technology, 2nd Edn. Boca Raton: CRC Press (2006).

[18] Hachana Y. Arabian mare's milk characterisation and clotting ability. J Food Sci Technol. (2020) 59:1840–6. 10.1007/s13197-021-05196-0 35531404 PMC9046472

[19] Csapó-Kiss Z Stefler J Martin T Makray S Csapó J. Composition of mares' colostrum and milk: protein content, amino acid composition, and contents of macro and micro-elements. Int Dairy J. (1995) 5:403–15. 10.1016/0958-6946(94)00014-G

[20] Kushugulova A Kozhakhmetov S Sattybayeva R Nurgaziyeva A Ziyat-Zhumagazy A Yadav H. Mare's milk as a prospective functional product. Funct Foods Health Dis. (2018) 8:537–43. 10.31989/ffhd.v8i11.528

[21] Fotschki J Szyc AM Laparra JM Markiewicz LH Wróblewska B. Immunomodulating properties of horse milk administered to mice sensitized to cow milk. J Dairy Sci. (2016) 99:9395–404. 10.3168/jds.2016-11499 27771084

[22] Andersen JH Osbakk SA Vorland LH Traavik T Gutteberg TJ. Lactoferrin and cyclic lactoferricin inhibit the entry of human cytomegalovirus into human fibroblasts. Antiviral Res. (2001) 51:141–9. 10.1016/S0166-3542(01)00146-2 11431038

[23] Pieszka M Łuszczyński J Zamachowska M Augustyn R Długosz B Hedrzak M. Is mare milk an appropriate food for people? A review. Ann Anim Sci. (2016) 16:33–51. 10.1515/aoas-2015-0041

[24] Benkerroum N. Antimicrobial activity of lysozyme with special relevance to milk. Afr J Biotechnol. (2008) 7:4856–67. 10.5897/AJB08.072

[25] Guri A Paligot M Crèvecoeur S Piedboeuf B Claes J Daube G. *In vitro* screening of mare's milk antimicrobial effect and antiproliferative activity. FEMS Microbiol Lett. (2016) 363:fnv234. 10.1093/femsle/fnv234 26656278

[26] Mariani P Summer A Martuzzi F Formaggioni P Sabbioni A Catalano AL. Physicochemical properties, gross composition, energy value, and nitrogen fractions of Halflinger nursing mare milk throughout six lactation months. Anim Res. (2001) 50:415–25. 10.1051/animres:2001140

[27] Kücükcetin A Yaygin H Hinrichs J Kulozik U. Adaptation of bovine milk towards mares' milk composition by means of membrane technology for koumiss manufacture. Int Dairy J. (2003) 13:945–51. 10.1016/S0958-6946(03)00143-2

[28] Pagliarini E Solaroli G Peri C. Chemical and physical characteristics of mare's milk. Ital J Food Sci. (1993) 4:323–32.

[29] Fusco V Chieffi D Fanelli F Logrieco AF Cho GS Kabisch J. Microbial quality and safety of milk and milk products in the 21st century. Compr Rev Food Sci Food Saf. (2020) 19:2013–49. 10.1111/1541-4337.12568 33337106

[30] Hogenboom JA Pellegrino L Sandrucci A Rosi V D'Incecco P. Invited review: hygienic quality, composition, and technological performance of raw milk obtained by robotic milking of cows. J Dairy Sci. (2019) 102:7640–54. 10.3168/jds.2018-16013 31255272

[31] Friman G Ilbäck NG Beisel WR. Effects of *Streptococcus pneumoniae, Salmonella typhimurium*, and *Francisella tularensis* infections on oxidative, glycolytic, and lysosomal enzyme activity in red and white skeletal muscle in the rat. Scand J Infect Dis. (1984) 16:111–9. 10.3109/13813458409068416 6320357

[32] Smagulov S. Osteometric study of some structure peculiarities of metapodium bones in the Adaev horse. Trudy Instituta Eksp Biologii Akad nauk Kazakhskoi SSR. (1986) 8:143–51.

[33] Dikko M Bello SO Chika A Mungadi IA Sarkingobir Y. Effect of tamsulosin use on plasma insulin status in benign prostatic hyperplasia patients in Sokoto, Nigeria. J Appl Sci Environ Manage. (2020) 24:543–8. 10.4314/jasem.v24i4.1

[34] Cais-Sokolińska D Teichert J Gawałek J. Foaming and other functional properties of freeze-dried mare's milk. Foods. (2023) 12:2274. 10.3390/foods12112274 37297518 PMC10252716

[35] Hill D Sugrue I Arendt E Hill C Stanton C Ross RP. Recent advances in microbial fermentation for dairy and health. F1000Res. (2017) 6:751. 10.12688/f1000research.10896.1 28649371 PMC5464223

[36] Marsh AJ Hill C Ross RP Cotter PD. Fermented beverages with health-promoting potential: past and future perspectives. Trends Food Sci Technol. (2014) 38:113–24. 10.1016/j.tifs.2014.05.002

[37] Francis FJ. Quality as influenced by color. Food Qual Prefer. (1995) 6:149–55. 10.1016/0950-3293(94)00026-R

[38] Bierzuńska P Cais-Sokolińska D Yigit A. Storage stability of texture and sensory properties of yogurt with the addition of polymerized whey proteins. Foods. (2019) 8:548. 10.3390/foods8110548 31689896 PMC6915489

[39] International Organization for Standardization. Milk and Milk Products—Guidance on Sampling. IDF Bulletin N° 42. Brussels, Belgium: International Dairy Federation (2008).

[40] International Organization for Standardization. Milk—determination of freezing point—thermistor cryoscope method (Reference method). Geneva, Switzerland: International Organization for Standardization (2009).

[41] Cais-Sokolińska D Danków R Bierzuńska P Kaczyński ŁK Chudy S Teichert J. Freezing point and other technological properties of milk of the Polish Coldblood horse breed. J Dairy Sci. (2018) 101:9637–46. 10.3168/jds.2018-15012 30197135

[42] Kristo E Biliaderis CG Tzanetakis N. Modelling of rheological, microbiological, and acidification properties of a fermented milk product containing a probiotic strain of *Lactobacillus paracasei*. Int Dairy J. (2003) 13:517–28. 10.1016/S0958-6946(03)00074-8

[43] Gaiani C Boyanova P Hussain R Murrieta Pazos I Karam MC Burgain J. Morphological descriptors and colour as a tool to better understand rehydration properties of dairy powders. Int Dairy J. (2011) 7:462–9. 10.1016/j.idairyj.2011.02.009

[44] Mainville I Montpetit D Durand N Farnworth ER. Deactivating the bacteria and yeast in kefir using heat treatment, irradiation, and high pressure. Int Dairy J. (2001) 11:45–9. 10.1016/S0958-6946(01)00038-3

[45] Gustaw W Kozioł J Radzki W Skrzypczak K Michalak-Majewska M Sołowiej B. The effect of addition of selected milk protein preparations on the growth of *Lactobacillus acidophilus* and physicochemical properties of fermented milk. Acta Sci Pol Technol Aliment. (2016) 15:29–36. 10.17306/J.AFS.2016.1.3 28071036

[46] Li C Li W Chen X Feng M Rui X Jiang M. Microbiological, physicochemical, and rheological properties of fermented soymilk produced with exopolysaccharide (EPS)-producing lactic acid bacteria strains. LWT Food Sci Technol. (2014) 57:477–85. 10.1016/j.lwt.2014.02.025

[47] Puerari C Magalhães KT Schwan RF. New cocoa pulp-based kefir beverages: microbiological, chemical composition, and sensory analysis. Food Res Int. (2012) 48:634–40. 10.1016/j.foodres.2012.06.005

[48] Malacarne M Martuzzi F Summer A Mariani P. Protein and fat composition of mare's milk: some nutritional remarks with reference to human and cow's milk. Int Dairy J. (2002) 12:869–77. 10.1016/S0958-6946(02)00120-6

[49] Caroprese M Albenzio M Marino R Muscio A Zezza T Sevi A. Behavior, milk yield, and milk composition of machine- and hand-milked Murgese mares. J Dairy Sci. (2007) 90:2773–7. 10.3168/jds.2006-603 17517717

[50] Radzki RP Bieńko M Polak P Szkucik K Ziomek M Ostapiuk M. Is the consumption of snail meat actually healthy? An analysis of the osteotropic influence of snail meat as a sole source of protein in growing rats. J Anim Physiol Anim Nutr. (2018) 102:e885–91. 10.1111/jpn.12851 29218776

[51] Nwoha PU. The blood constituents of gossypol-treated, protein-malnourished Wistar rats. Contraception. (1995) 52:249–54. 10.1016/0010-7824(95)00188-G 8605784

[52] Park YW Nam MS. Bioactive peptides in milk and dairy products: a review. Korean J Food Sci Anim Resour. (2015) 35:831–40. 10.5851/kosfa.2015.35.6.831 26877644 PMC4726964

[53] Westerterp-Plantenga MS Lemmens SG Westerterp KR. Dietary protein—its role in satiety, energetics, weight loss, and health. Br J Nutr. (2012) 108:S105–12. 10.1017/S0007114512002589 23107521

[54] Halton TL Hu FB. The effects of high protein diets on thermogenesis, satiety, and weight loss: a critical review. J Am Coll Nutr. (2004) 23:373–85. 10.1080/07315724.2004.10719381 15466943

[55] Slavin J. Dietary fiber and body weight. Nutr. (2013) 29:411–8. 10.1016/j.nut.2004.08.018 15797686

[56] Cummings JH Stephen AM. Carbohydrate terminology and classification. Eur J Clin Nutr. (2007) 61:S5–S18. 10.1038/sj.ejcn.1602936 17992187

[57] Stanhope KL. Sugar consumption, metabolic disease, and obesity: the state of the controversy. Crit Rev Clin Lab Sci. (2016) 53:52–67. 10.3109/10408363.2015.1084990 26376619 PMC4822166

[58] Xiao YH Zhan CL Li JJ Wu J Li XC Zheng WL. [Comparison of serum biochemistry between specific pathogen-free and conventional aged Wistar rats]. Di Yi Jun Yi Da Xue Xue Bao. (2004) 24:733–5. [In Chinese]. 15257887

[59] Chiavolini D Pozzi G Ricci S. Animal models of *Streptococcus pneumoniae* disease. Clin Microbiol Rev. (2008) 21:666–85. 10.1128/CMR.00012-08 18854486 PMC2570153

[60] Park SJ Lee HJ Kim CK Kim IH Kim MG Lee MG. Toxicity study of *Streptococcus pneumoniae* vaccine administered subcutaneously in rats. Toxicol Res. (2011) 27:111–8. 10.5487/TR.2011.27.2.111 24278559 PMC3834373

[61] Kosyreva AM Kirpichnikov MP Khasanova NZ Alekseeva OP Kolyadko VN Avakyan GG. Sex differences of inflammatory and immune response in pups of Wistar rats with SIRS. Sci Rep. (2020) 10:15884. 10.1038/s41598-020-72537-y 32985516 PMC7522713

[62] Steinberg P van der Voet H Goedhart PW Kleter GA Kok EJ Kuiper HA. Lack of adverse effects in subchronic and chronic toxicity/carcinogenicity studies on the glyphosate-resistant genetically modified maize NK603 in Wistar Han RCC rats. Arch Toxicol. (2019) 93:1095–139. 10.1007/s00204-019-02400-1 30756133 PMC7261740

[63] Rahman MM Bhuiyan MJH Alam MN Uddin MM Haque ME. Hematological and serum biochemical reference values for Wistar rats. Lab Anim Res. (2024) 40:25–32.38898483

[64] Dmitrieva N Simonova O Lebedeva E Smirnova O. Systemic inflammation enhances immune cell mobilization and hematopoietic response. Front Immunol. (2021) 12:595722.33708198

[65] Gao Y Souza-Fonseca-Guimaraes F Bald T Ng SS Young A Ngiow SF. Cytokine and chemokine profiles regulate myelopoiesis and cellular mobilization during inflammation. Front Immunol. (2022) 13:918051.

[66] Takeuchi O Akira S. Pattern recognition receptors and inflammation: activation of PRRs by DAMPs and PAMPs promotes immune responses. Nat Rev Immunol. (2018) 18:580–98.

[67] Canny SP Orozco SL Thulin NK Hamerman JA. Immune mechanisms in inflammatory anemia. Annu Rev Immunol. (2023) 41:405–29. 10.1146/annurev-immunol-101320-125839 36750316 PMC10367595

[68] Cieslik E Kopec A Piatkowska E. Mare's milk as a prospective functional product. J Nutr Health Aging. (2019) 23:484–9. 10.1007/s12603-019-1195-0

